# Establishing an atherosclerosis diagnostic model based on WGCNA and machine learning algorithms with key genes in cholesterol metabolism and ferroptosis, and revealing the regulatory role of HMOX1 in cellular ferroptosis

**DOI:** 10.3389/fcvm.2026.1715946

**Published:** 2026-03-12

**Authors:** Zengguang Fan, Caihui Liu, Yiwen Liu, Zijian Hong, Jianming Zhong, Bei Yang, Ye Yuan

**Affiliations:** 1 Cardiovascular Medicine Department, Affiliated Hospital of Jiangxi University of Chinese Medicine, Nanchang, Jiangxi, China; 2 Jiangxi University of Chinese Medicine, Nanchang, Jiangxi, China; 3 Endocrine Department, Affiliated Hospital of Jiangxi University of Chinese Medicine, Nanchang, Jiangxi, China

**Keywords:** atherosclerosis, cholesterol metabolism, ferroptosis, machine learning, WGCNA

## Abstract

**Background:**

As the primary pathological basis for cardiovascular diseases, atherosclerosis (AS) arises from pathogenesis closely linked to dysregulated cholesterol metabolism and ferroptosis. This study seeks to develop an AS diagnostic model and identify potential biomarkers.

**Methods:**

AS-related transcriptomic datasets were obtained from the GEO database. Differentially expressed cholesterol metabolism- and ferroptosis-related genes (DE-CM-FRGs) were screened by integrating WGCNA module genes, AS-related differentially expressed genes, cholesterol metabolism-related genes, and ferroptosis-related genes. Consensus clustering was performed to subtype AS patients. Hub genes were refined using three machine learning algorithms: Least Absolute Shrinkage and Selection Operator (LASSO), Support Vector Machine-Recursive Feature Elimination (SVM-RFE) and Boruta. A logistic regression diagnostic model based on filtered genes was established and evaluated with ROC curves. A nomogram was constructed and evaluated through calibration, decision, and impact curves, followed by building a diagnostic gene-based regulatory network. Single-cell RNA sequencing analyzed HMOX1-expressing cells. *In vitro*, HMOX1 knockdown effects on proliferation, ROS, MDA, iron content, and mRNA expression of SLC7A11, GPX4, and ACSL4 were assessed in ox-LDL-induced THP-1 cells.

**Results:**

The identified five core feature genes (CD36, DPP4, HMOX1, IL1B, NFIL3) exhibited robust diagnostic relevance and auxiliary discriminant value across both training and validation sets. The diagnostic model based on these five genes exhibited strong discriminatory ability in both sets. Regulatory network analysis revealed interactions between the diagnostic genes and transcription factors, miRNAs, and compounds. HMOX1 knockdown suppressed ox-LDL-induced THP-1 cell proliferation, lowered intracellular ROS, MDA, and iron levels, upregulated GPX4 and SLC7A11 expression, and downregulated ACSL4.

**Conclusion:**

By systematically identifying key genes in AS-associated cholesterol metabolism and ferroptosis, this study constructs a robust diagnostic model and identifies potential biomarkers and therapeutic targets for AS diagnosis.

## 1 Introduction

Atherosclerosis (AS) is a critical pathological foundation for cardiovascular and cerebrovascular diseases, characterized primarily by lipid deposition in the arterial wall, accompanied by smooth muscle cell proliferation and fibrous matrix accumulation, gradually forming atherosclerotic plaques (1). Upon unstable plaques rupturing, they trigger platelet aggregation and thrombosis, potentially leading to vascular stenosis or occlusion and acute cardiovascular events (2). Due to its high incidence, disability, and mortality rates, and a trend towards earlier onset with modern dietary and exercise habits (3), early detection, diagnosis, and treatment are paramount in current prevention and control efforts.

AS development is driven by key biological processes, including lipid metabolism disorders, inflammation, oxidative stress, and cell death (4). Cholesterol metabolism imbalance is a critical risk factor. AS arises from lipid metabolism dysregulation, which underlies the pathological accumulation of cholesterol in arterial walls and subsequent plaque formation. Concurrently, elevated plasma cholesterol levels exacerbate AS progression (5, 6). Thus, effective regulation of cellular and systemic cholesterol metabolism is critical for AS prevention. Furthermore, circulating cholesterol level homeostasis is closely linked to AS pathogenesis. One clinical study shows that controlling high cholesterol levels can effectively prevent the occurrence of atherosclerotic cardiovascular disease (ASCVD) (7). Apolipoprotein B (ApoB)-containing lipoproteins are key drivers of ASCVD, with LDL being the predominant type (8). Reducing ApoB concentration decreases circulating atherogenic lipoprotein particles, thereby lowering LDL-C to prevent ASCVD (9, 10).

As a regulated form of programmed cell death, ferroptosis is dependent on reactive oxygen species accumulation and iron overload. This iron-mediated, lipid peroxidation-driven cell death process offers a new perspective on AS nature (11). Emerging evidence links ferroptosis to cardiovascular diseases, including AS and cardiomyopathy (12). Bai *et al*. (13) elucidated that ferroptosis accelerates AS progression, while its inhibition mitigates the condition by reducing lipid peroxidation and endothelial dysfunction. Additionally, inactivation of GPX4, a key ferroptosis gene, may promote fundamental plaque formation processes, suggesting ferroptosis inhibition as an underlying therapeutic strategy (14).

This study integrates weighted gene co-expression network analysis (WGCNA) with multiple machine learning algorithms, combining AS-related differentially expressed genes (DEGs) with key genes related to cholesterol metabolism and ferroptosis to construct a logistic regression diagnostic model. Its predictive performance was evaluated via receiver operating characteristic (ROC) curves, calibration curves, and DCA. Finally, through immune infiltration analysis, regulatory network construction, and single-cell expression validation, we comprehensively assessed the biological functions and clinical potential of the key genes. This provides potential biomarkers and diagnostic tools for early AS diagnosis and subtyping, holding pivotal theoretical value and clinical translation possibility.

## 2 Methods

### 2.1 Data collection and preprocessing

AS-related datasets were downloaded from the Gene Expression Omnibus (GEO, https://www.ncbi.nlm.nih.gov/geo/), including GSE100927 as the training set (35 controls, 69 AS cases), GSE43292 as the validation set (32 controls, 32 AS cases), and GSE155512 for single-cell RNA sequencing (scRNA-seq).

The GSE100927 dataset was preprocessed in R. Raw probe signals were normalized against a virtual median chip using the locally weighted scatter plot smoothing method, duplicate probes were averaged, and low-intensity probes were removed. The GSE43292 dataset was processed with Expression Console v1.1, applying the default robust multi-array average (RMA) method for background correction, quantile normalization, and log transformation. Although the two datasets differed in RMA implementation details, both followed the RMA normalization framework. To improve comparability across datasets, all expression data were uniformly converted to the log2 scale and subjected to stringent quality control.

The GSE155512 dataset contains single-cell transcriptomic data from three human atherosclerotic carotid arteries. It was analyzed using the “Seurat” package in R (v4.4.2). The raw data were normalized and scaled with the “NormalizeData” and “ScaleData” functions. The top 3,000 highly variable genes were selected with the “FindVariableFeatures” function for principal component analysis (PCA). To mitigate batch effects and integrate samples into a shared low-dimensional space, the “RunHarmony” function from the “Harmony” package was applied. The number of principal components (PCs) for downstream analysis was determined based on the variance explained by each PC, applying the following criteria: (1) the cumulative explained variance exceeded 80%, with each additional PC contributing less than 5%; (2) the drop in variance between consecutive PCs fell below 0.1 for the first time. The top 23 PCs were used for UMAP dimensionality reduction and visualization. Unsupervised clustering was performed with the “FindNeighbors” and “FindClusters” functions (resolution = 0.5) to construct a cell atlas.

Cholesterol metabolism-related genes (CMRGs) were sourced from the Molecular Signatures Database (https://www.gsea-msigdb.org/gsea/msigdb/index.jsp), including five gene sets: Hallmark cholesterol homeostasis, GOBP regulation of cholesterol, WP cholesterol metabolism, WP cholesterol biosynthesis, and Reactive cholesterol biosynthesis. Concurrently, genes related to the cholesterol metabolism pathway from the Kyoto Encyclopedia of Genes and Genomes (KEGG) (map04979) were also included. After merging and deduplication, 170 CMRGs were obtained for subsequent analysis.

Ferroptosis-related genes (FRGs) were obtained from the Ferroptosis Database (http://www.zhounan.org/ferrdb), including Driver, Suppressor, and Marker genes. After deduplication, 484 genes were included.

### 2.2 Identification of DEGs

The “limma” package (15) was implemented to identify DEGs between AS and control samples in GSE100927 (|log2FC| > 1, adjusted *p*-value < 0.05).

### 2.3 WGCNA

The “WGCNA” package (16) was applied to the expression matrix of AS and control samples in GSE100927. Genes exhibiting expression variance within the top 25% were selected as input for WGCNA, followed by correlation analysis with sample groupings to identify phenotype-associated modules. A soft-thresholding power *β* = 14 was chosen to achieve a scale-free network. Gene co-expression modules were identified by constructing a hierarchical clustering dendrogram using the blockwiseModules function, followed by dynamic tree cutting (minModuleSize = 50). The resulting modules were visually distinguished by color. Subsequently, the “pheatmap” package (17) was employed to visualize correlations between phenotypic data and module eigengenes, enabling the selection of modules with significant associations.

### 2.4 Identification of differentially expressed cholesterol metabolism- and ferroptosis-related genes (DE-CM-FRGs)

Intersection genes were identified among WGCNA module genes, DEGs, FRGs, and CMRGs, defining key DE-CM-FRGs for further analysis.

### 2.5 Consensus clustering of DE-CM-FRGs and immune infiltration analysis

Unsupervised clustering was performed to stratify patients into molecular subtypes based on the expression profiles of DE-CM-FRGs. The consensus clustering algorithm was implemented using the “ConsensusClusterPlus” R package (18), with 1,000 iterations to evaluate clustering stability and determine the optimal cluster number for subsequent analyses. PCA was conducted to analyze AS patient samples in the training set using the prcomp function and visualized with the “factoextra” package (19). To further compare the expression discrepancies of DE-CM-FRGs among distinct subtypes, a boxplot was plotted utilizing the “Ggpubr” package (20). The single-sample Gene Set Enrichment Analysis (ssGSEA) was applied to evaluate the enrichment degree of immune-related cells and immune-related pathways in each subtype. The Wilcoxon rank-sum test was used to evaluate differences in ssGSEA scores across molecular subtypes, with *p* < 0.05 considered statistically significant.

### 2.6 KEGG and gene ontology (GO) enrichment analyses of DE-CM-FRGs

GO and KEGG enrichment analyses for DE-CM-FRGs were performed employing the “clusterProfiler” package (21) (significance thresholds pvalueCutoff = 0.05, qvalueCutoff = 0.05). Results were visualized via the “enrichplot” package (22).

### 2.7 Machine learning for Hub gene selection

To identify optimal diagnostic feature genes, three machine learning algorithms were applied to the DE-CM-FRGs: least absolute shrinkage and selection operator (LASSO) regression via the “glmnet” package (23), support vector machine-recursive feature elimination (SVM-RFE) via the “caret” package (24), and the Boruta algorithm via the “Boruta” package (25). The intersection of genes selected by all three algorithms was defined as the key hub genes.

A logistic regression diagnostic model was established by leveraging the “rms” package (26). The model formula was: logit(P) = *β*0 + *β*1*Gene1 + *β*2*Gene2  + … + *β*n*Genen [logit(P): the log-odds of the event (disease), *β*0: the intercept term, *β*1 and *β*2: the regression coefficients for each gene]. To evaluate the discriminative power of the model, the area under the ROC (AUC) was adopted as the quantitative indicator. ROC curves were generated, and AUC values were computed utilizing the “pROC” R package (27).

### 2.8 Construction and validation of the nomogram

A nomogram was constructed using the “rms” package, with model calibration assessed via calibration curves to evaluate prediction accuracy. Furthermore, the “rmda” package (28) was employed to perform decision curve analysis (DCA) and generate clinical impact curves, quantifying net benefit across threshold probabilities, thereby determining the model's clinical utility and decision-making value.

### 2.9 Correlation between model genes and immune cells

To systematically evaluate immune microenvironment differences between subtypes (cluster 1 vs. cluster 2), immune cell infiltration was assessed using ssGSEA and the CIBERSORT algorithm. ssGSEA was performed with the “GSVA” R package. Immune cell infiltration was quantified using feature gene sets from literature (29) under parameters kcdf = “Gaussian” and abs.ranking = TRUE. Between-group differences were tested by the Wilcoxon rank-sum test with Benjamini-Hochberg FDR correction (significance threshold *p* < 0.05; Control = 35, AS = 69). The CIBERSORT deconvolution algorithm (LM22 signature matrix, 100 permutations, QN = TRUE) was used to estimate immune cell composition. Only samples with *p* < 0.05 were retained for subsequent analysis (Control = 34, AS = 69). Differences in immune cell proportions between groups were assessed with the Kruskal–Wallis test (*p* < 0.05 considered statistically significant).

### 2.10 Construction of transcription factor (TF)-miRNA regulatory network

The TF-miRNA regulatory network for the hub genes was constructed using NetworkAnalyst (https://www.networkanalyst.ca/) based on interactions from the RegNetwork database. Nodes with a degree >1 were retained to simplify the structure. The network was visualized employing Cytoscape 3.10.0.

### 2.11 Protein-Chemical interaction analysis for Key genes

By leveraging NetworkAnalyst and data from the Comparative Toxicogenomics Database (CTD, https://ctdbase.org/), interaction networks between proteins encoded by the key genes and chemicals were predicted and visualized with Cytoscape 3.10.0.

### 2.12 Single-cell level expression analysis of model genes

Model gene expression was analyzed at single-cell resolution on the Single Cell Portal (https://singlecell.broadinstitute.org/single_cell). A human carotid artery disease dataset (30) was employed to compare model gene expression between disease and normal control groups (1 control, 2 disease).

Meanwhile, a mouse dataset (31) was used to examine expression changes of homologous genes in AS progression (*n* = 1) vs. regression (*n* = 1) groups, providing cross-species validation of their expression trends during AS onset and progression. Experiments were conducted in Cx3cr1^CreERT2−IRES−YFP/+^; Rosa26^floxed−tdTomato/+^ mice. AS was induced with AAV-mPCSK9 and fed with 18 weeks of Western-type high-fat diet, with CX3CR1^+^ precursor cells labeled by Tamoxifen. Mice were then divided into a progression group (continued Western diet for 2 weeks to maintain plaque progression) and a regression group (switched to chow diet and intraperitoneal ApoB-ASO injection at 50 mg/kg twice weekly for 2 weeks to lower plasma ApoB and induce plaque regression). After aortic arch digestion, CD11b^+^ TdTomato^+^ cells were isolated by FACS and subjected to scRNA-seq on the 10X Genomics platform. A total of 3,157 cells in the progression group and 2,198 cells in the regression group were analyzed, with 4 mice per group and 3 independent experimental replicates. Statistical analysis was performed using Student's *t*-test, and results are presented as mean ± SEM. Furthermore, the distribution of the key gene HMOX1 across different cell types was examined in the GSE155512 dataset.

### 2.13 Cell culture

THP-1-derived macrophages (TIB-202, ATCC, USA) were cultured in 1,640 medium containing 10% fetal bovine serum and 1% penicillin and passaged every 2–3 days. Differentiation into macrophages was induced by stimulating THP-1 cells with 50 nM phorbol myristate acetate (16561-29-8, Merck, USA) for 48 h. Then, 100 μg/mL oxidized low-density lipoprotein (ox-LDL) (308068-14-6, Merck, USA) was applied for 48 h to form foam-like macrophages.

### 2.14 Cell transfection

Using the Lipofectamine 2,000 reagent kit (11668019, Thermo Fisher, USA), the small interfering RNA targeting HMOX1 (si-HMOX1) and the negative control (si-NC) (Hanbio, China) were transfected into ox-LDL-treated macrophages for 24 h before subsequent experiments.

### 2.15 Quantitative real-time PCR (qRT-PCR)

Total RNA was extracted by leveraging Trizol reagent (15596018CN, Thermo Fisher, USA), followed by concentration and purity measurement via a NanoDrop spectrophotometer. cDNA was synthesized by implementing HiScript III RT SuperMix (R323-01, Vazyme, China). qRT-PCR was conducted with SYBR Green qPCR kit (CL132-01, Vazyme, China) on a Step-One Plus Real-Time PCR System. Primer sequences are provided in Table 1.

**Table 1:** Primers for qRT-PCR.

Gene	Forward primer (5′-3′)	Reverse Primer (5′-3′)
HMOX1	AAGACTGCGTTCCTGCTCAA	GGGGGCAGAATCTTGCACT
SLC7A11	TGCCCAGATATGCATCGTCC	TGTTCTGGTTATTTTCTCCGACA
GPX4	GAAGATCCAACCCAAGGGCA	GACGGTGTCCAAACTTGGTG
ACSL4	GAGAAAAAGAGGACATTTGCGT	CAGCCAAGGCAGTTCAATCTT
GAPDH	AATGGGCAGCCGTTAGGAAA	GCCCAATACGACCAAATCAGAG

### 2.16 CCK-8 cell viability assay

HUVECs from distinct treatment groups were inoculated in 96-well plates (5 × 10^3^ cells/well). After treatments, 10 μL of CCK-8 reagent (C0037, Beyotime, China) was added to each well and incubated for 2 h. Absorbance at 450 nm was measured by a microplate reader to assess cell viability.

### 2.17 Detection of intracellular reactive oxygen Species (ROS)

Intracellular ROS was detected with the DCFH-DA fluorescent probe (S0033S, Beyotime, China). Treated cells were incubated with DCFH-DA diluted in serum-free medium at 37 °C in the dark for 20 min, washed, and immediately observed under a confocal microscope (excitation light 488 nm, emission light 500–530 nm). Random fields were selected for imaging. Fluorescence intensity was quantified with ImageJ to compare between-group differences.

### 2.18 Determination of intracellular malondialdehyde (MDA)

Lipid peroxidation was detected with the MDA detection kit (S0131S, Beyotime, China). Collected cells were completely lysed by repeated freezing and thawing. The resulting lysate was centrifuged at 10,000 g for 10 min at 4 °C. The optical density (OD) value corresponding to the MDA content in the supernatant was determined according to the kit instructions.

### 2.19 Determination of intracellular iron Ion content

The intracellular iron ion content was determined with the iron ion (Fe^2+^) detection kit (ab83366, Abcam, UK). Cells from each group were lysed, and the supernatant was obtained after centrifugation. The iron ion concentration of each group was determined based on the OD value at 560 nm.

### 2.20 Statistical analysis

Experimental data were statistically analyzed in GraphPad Prism 10.1.2 and R (version 4.4.2). Continuous variable data were expressed as mean ± standard deviation from at least three independent repeated experiments. Between-group differences were compared with a *t*-test or the Wilcoxon rank sum test. A *p* < 0.05 was considered statistically significant.

## 3 Results

### 3.1 Identification and determination of DE-CM-FRGs

**Figure 1:**
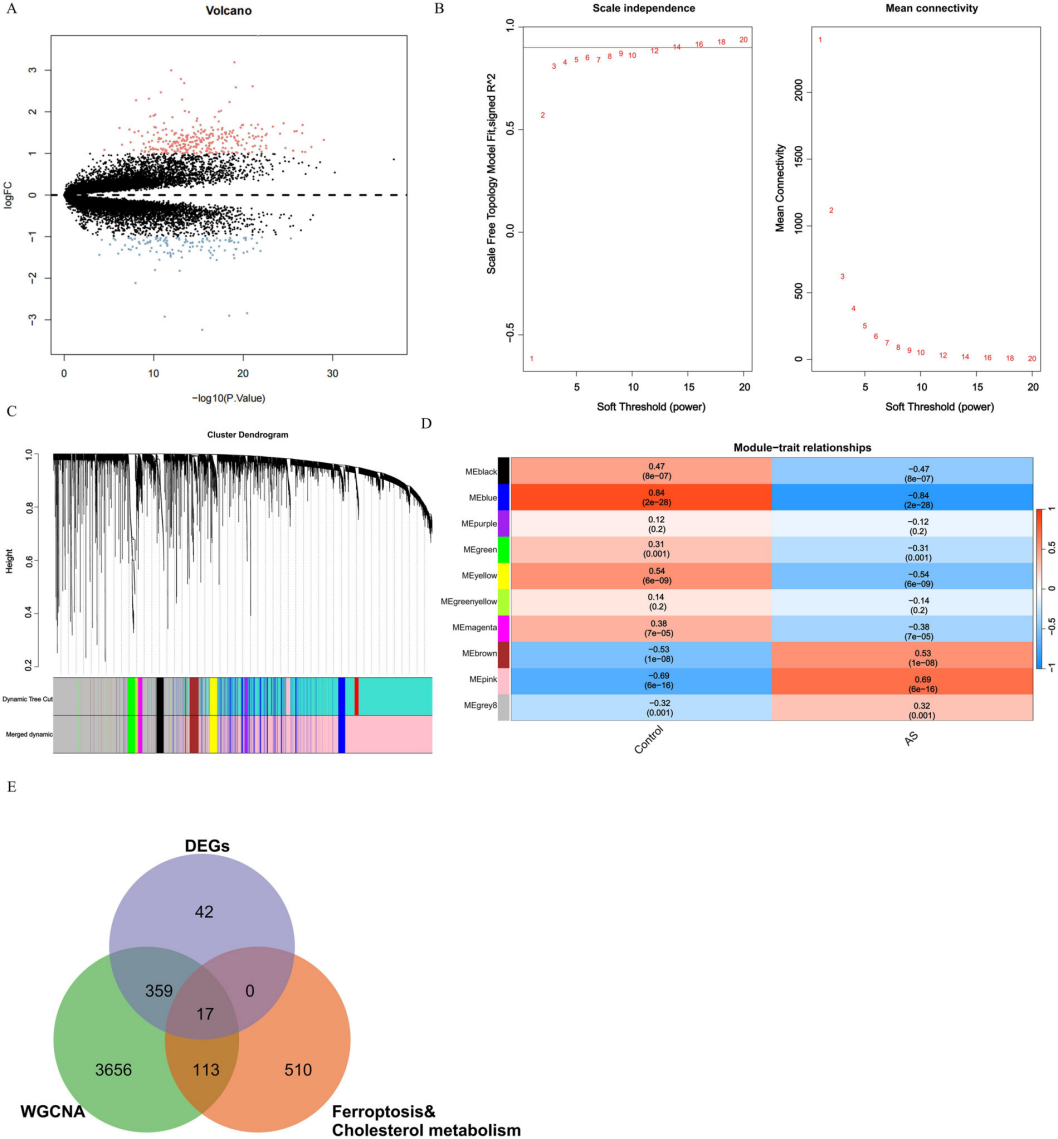
Identification and determination of DE-CM-FRGs. **(A)** Volcano plot of DEGs between AS and control samples in the GSE100927 dataset (35 Control, 69 AS), presenting their distribution of log_2_FC vs. statistical significance –log_10_ (P.value) based on differential expression analysis. **(B)** Selection of the soft threshold power with the “WGCNA” package based on the principles of scale independence and mean connectivity. **(C)** Construction of a weighted gene co-expression network and identification of distinct gene expression modules with the “WGCNA” package. **(D)** Heatmap depicting correlations between module eigengenes and clinical phenotypes (Control vs. AS) calculated with the “WGCNA” package and visualized with the “pheatmap” package. **(E)** Venn diagram analysis performed with the “VennDiagram” package to identify DE-CM-FRGs (intersection of AS-associated WGCNA module genes, DEGs in the GSE100927 dataset, FRGs, and CMRGs).

Differential expression analysis of AS and control samples in GSE100927 identified 418 DEGs (Supplementary Table S1, Figure 1A). Further application of the GSE100927 dataset for WGCNA analysis involved hierarchical clustering of these two samples within the dataset, resulting in a clustering tree structure that reflects the similarities among the samples. A scale-free network was constructed based on a soft threshold power *β* = 14 (Figure 1B). Subsequently, multiple co-expression modules were identified according to the extracted gene information in each module (minModuleSize = 50) (Figure 1C). Module-trait relationship analysis revealed that the pink and brown modules had the highest correlations with AS (Figure 1D). Intersecting genes from these two modules with DEGs, CMRGs, and FRGs yielded 17 key DE-CM-FRGs for further study (Supplementary Table S2, Figure 1E).

**Figure 2:**
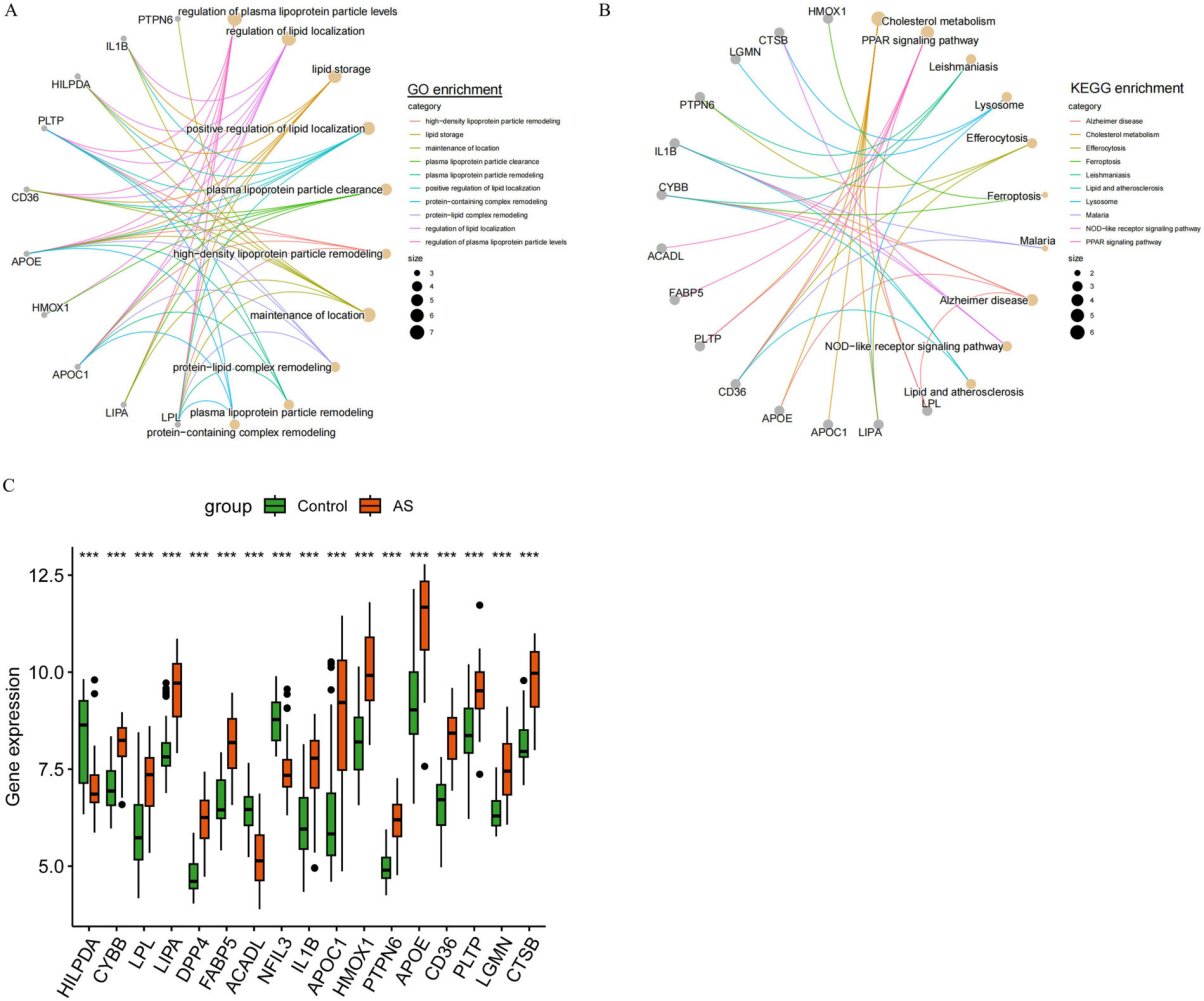
Enrichment analysis of DE-CM-FRGs. **(A,B)**: GO **(A)** and KEGG **(B)** enrichment analyses of DE-CM-FRGs with the “clusterProfiler” package. **(C)**: Differential expression analysis of DE-CM-FRGs between the Control and AS groups with the “limma” package. Statistical significance was assessed with the Wilcoxon rank-sum test, ***: *p* < 0.001. All analyses were conducted in R (version 4.4.2).

GO and KEGG enrichment analyses indicated these genes were primarily involved in lipid metabolism processes (e.g., regulation of plasma lipoprotein particle levels, lipid storage, plasma lipoprotein particle clearance) and metabolism pathways (e.g., Cholesterol metabolism, Lipid and atherosclerosis, PPAR signaling pathway) (Figures 2A,B). These enrichment results highlight the gene set's core role in cholesterol metabolism regulation. Crucially, as DE-CM-FRGs were derived by intersecting WGCNA module genes, DRGs, and FRGs, they not only participate in lipid metabolism but also form potential molecular hubs linking cholesterol metabolism to ferroptosis. This indicates that in AS, lipid metabolism disorders may cooperatively or interactively crossregulate with ferroptosis-associated cellular damage. To observe the alterations of these DE-CM-FRGs, we performed the expression analysis, which indicated that only HILPDA, ACADL, and NFIL3 were downregulated in AS, while the others were upregulated (Figure 2C).

### 3.2 AS subtype identification based on DE-CM-FRGs and immune microenvironment characteristics

**Figure 3:**
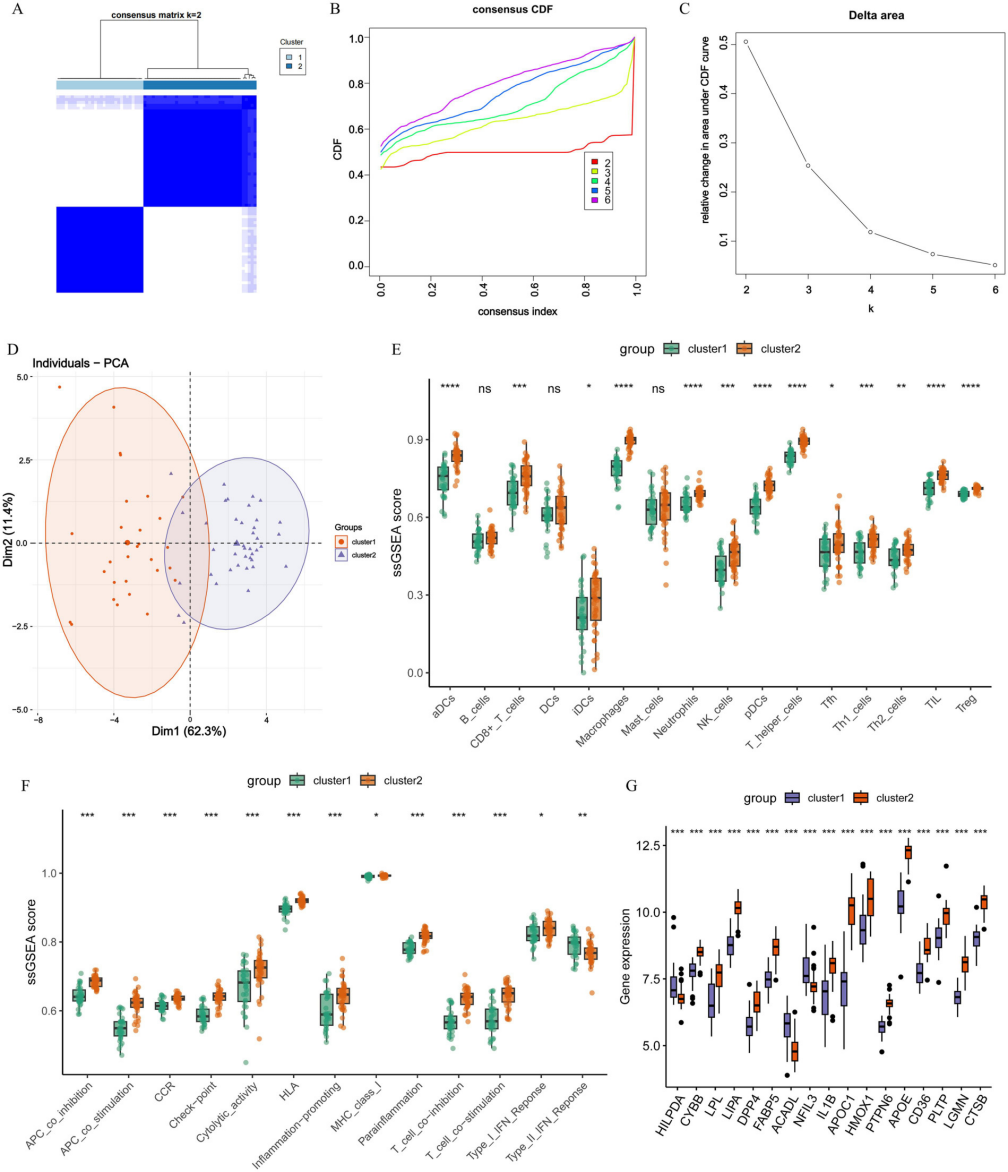
AS subtype identification based on DE-CM-FRGs and immune microenvironment characteristics. **(A)** Consistency clustering analysis of AS samples based on the DE-CM-FRGs expression profile with the “ConsensusClusterPlus”, resulting in two molecular subtypes. **(B)** Cumulative distribution function (CDF) curve. **(C)** Relative changes in the area under the CDF curve under different clustering numbers. **(D)** Verification of the clustering separation effect of the two molecular subtypes by principal component analysis (PCA) with the “prcomp” function. **(E,F)** Comparison of the immune cell infiltration **(E)** and the activity of immune function-related pathways **(F)** between the two subtypes by ssGSEA with the “GSVA” package. **(G)** Differential expression analysis of DE-CM-FRGs between the two AS molecular subtypes with the “limma” package. Statistical differences were assessed with the Wilcoxon rank sum test, *: *p* < 0.05, **: *p* < 0.01, ***: *p* < 0.001, ****: *p* < 0.0001. All analyses were conducted in R (version 4.4.2).

Consensus clustering based on the 17 DE-CM-FRG expression profiles stratified AS patients into two stable molecular subtypes (cluster 1 and cluster 2) (Figure 3A). To validate the stability of clustering results, cumulative distribution function (CDF) curves and the relative changes in the AUC were analyzed. The CDF curve demonstrated optimal stability at *k* = 2, while the AUC exhibited a pronounced decline when k exceeded 2. These findings collectively indicate that *k* = 2 represents the optimal number for subtyping (Figures 3B,C). As shown by PCA, the two subtypes were clearly separated, further validating the reliability of the clustering results (Figure 3D). ssGSEA revealed, compared with cluster 1, significantly higher infiltration of macrophages, neutrophils, and mast cells in cluster 2, along with enhanced activity in inflammatory responses like Cytolytic activity and Type I IFN Response, suggesting an immune-inflammatory microenvironment in cluster 2 (Figures 3E,F). Further observation demonstrated that DE-CM-FRG expression differed between subtypes, with HILPDA, ACADL, and NFIL3 lower in cluster 2, and the remaining genes higher, compared with cluster 1 (Figure 3G).

### 3.3 Machine learning-based screening of candidate hub genes and diagnostic value assessment

**Figure 4:**
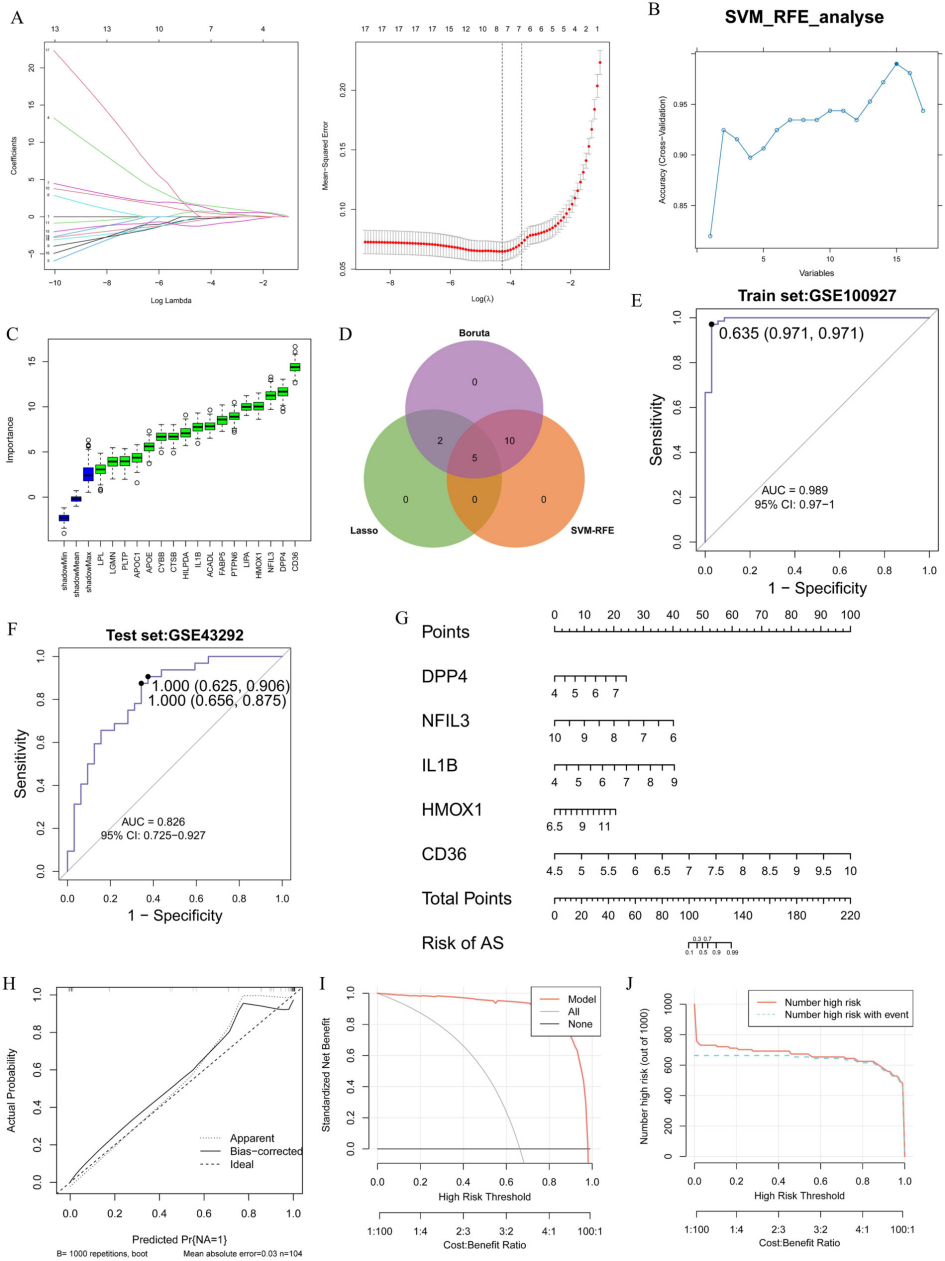
Machine learning-based screening of candidate hub genes and diagnostic value assessment. **(A)** Screening of candidate feature genes by LASSO regression based on DE-CM-FRGs with the “glmnet” package. **(B)** Refinement of candidate genes by SVM-RFE with the “e1071” and “caret” packages. **(C)** Assessment of gene importance by the Boruta algorithm for feature selection with the “Boruta” package. **(D)** Determination of key diagnostic hub genes by intersecting genes selected by the LASSO, SVM-RFE, and Boruta algorithms. **(E,F)** Construction of a logistic regression model based on the identified hub genes and evaluation of diagnostic performance via ROC curves in both the training **(E)** and validation **(F)** sets with the “pROC” package. **(G)** Development of a nomogram based on the logistic regression model with the “rms” package. **(H–J)** Assessment of the nomogram model through calibration curve **(H)**, decision curve analysis **(I)**, and impact curve **(J)** to evaluate prediction consistency, clinical net benefit, and model stability, respectively, with the “rms” and “rmda” packages. All analyses were conducted in R (version 4.4.2).

To identify diagnostic genes for AS, we performed feature selection on DE-CM-FRGs employing three distinct algorithms: LASSO, SVM-RFE, and Boruta. Through 10-fold cross-validation, the optimal *λ* value was determined to be 0.01390684, leading to the identification of 7 genes via LASSO (Figure 4A). Subsequently, SVM-RFE selected 15 genes (Figure 4B), while the Boruta algorithm filtered 17 candidate genes (Figure 4C). The intersection of these three sets yielded five key diagnostic genes: CD36, DPP4, HMOX1, IL1B, and NFIL3 (Figure 4D). The performance of a logistic regression model built with these genes was verified in the training set (AUC = 0.989, 95% CI: 0.97-1.00) and the validation set (AUC = 0.826, 95% CI: 0.725–0.927) through the ROC curve, indicating that the model had good accuracy (Figures 4E,F). To further verify the predictive performance of the model, we constructed a nomogram based on five diagnostic genes (Figure 4G). The results of the calibration curve, DCA, and impact curve all indicated that this nomogram had good predictive capabilities (Figures 4H–J).

### 3.4 Validation of key diagnostic genes

**Figure 5:**
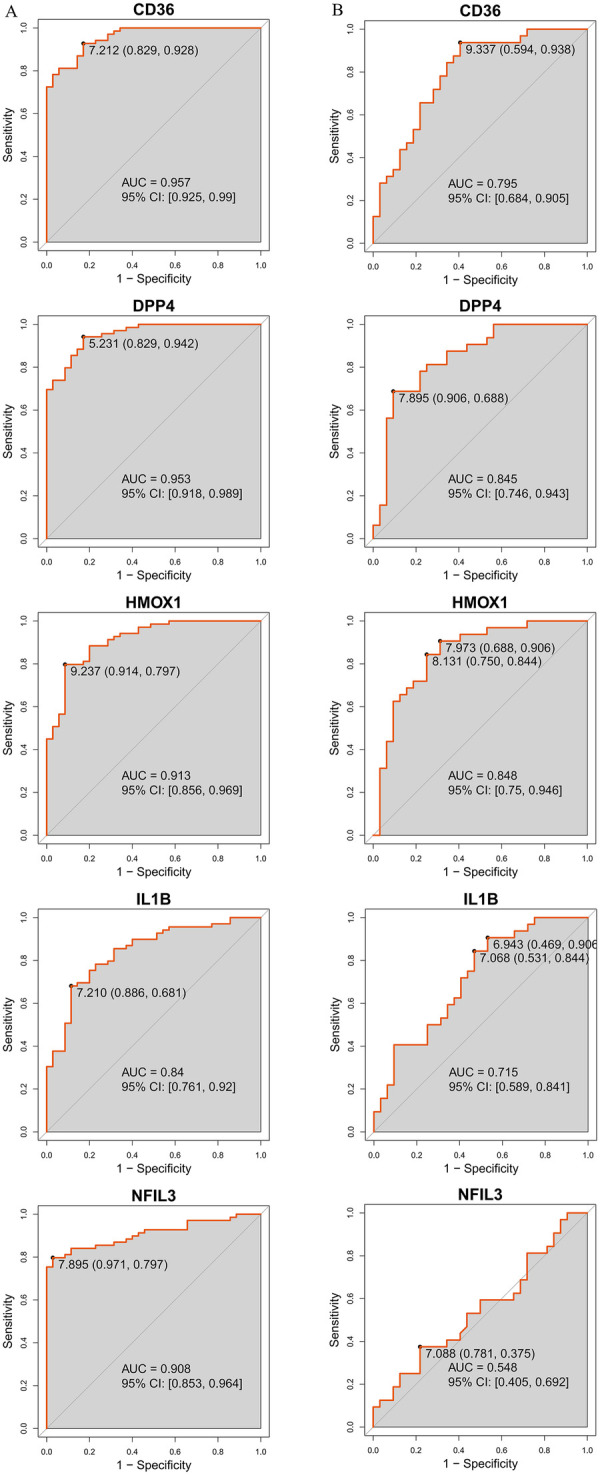
Validation of key diagnostic genes. **(A,B)** ROC curves of the key diagnostic genes in the training set **(A)** and validation set **(B)** based on the logistic regression model, with the area under the curve (AUC) calculated using the “pROC” package to evaluate the discriminatory ability of the key diagnostic genes for AS. All analyses were conducted in R (version 4.4.2).

The diagnostic efficacy of each key gene was evaluated by leveraging ROC curves with the calculation of AUC values. In the training set, AUC values for CD36, DPP4, HMOX1, IL1B, and NFIL3 were 0.957, 0.953, 0.913, 0.840, and 0.908, respectively (Figure 5A). In the validation set, the AUC values were 0.795, 0.845, 0.848, 0.715, and 0.548, respectively (Figure 5B). Although NFIL3's AUC was lower in the validation set, the other four genes maintained good diagnostic performance (AUC > 0.7), indicating their robustness as potential AS biomarkers.

### 3.5 Gene-TF regulatory network and protein-chemical interaction networks for model genes

**Figure 6:**
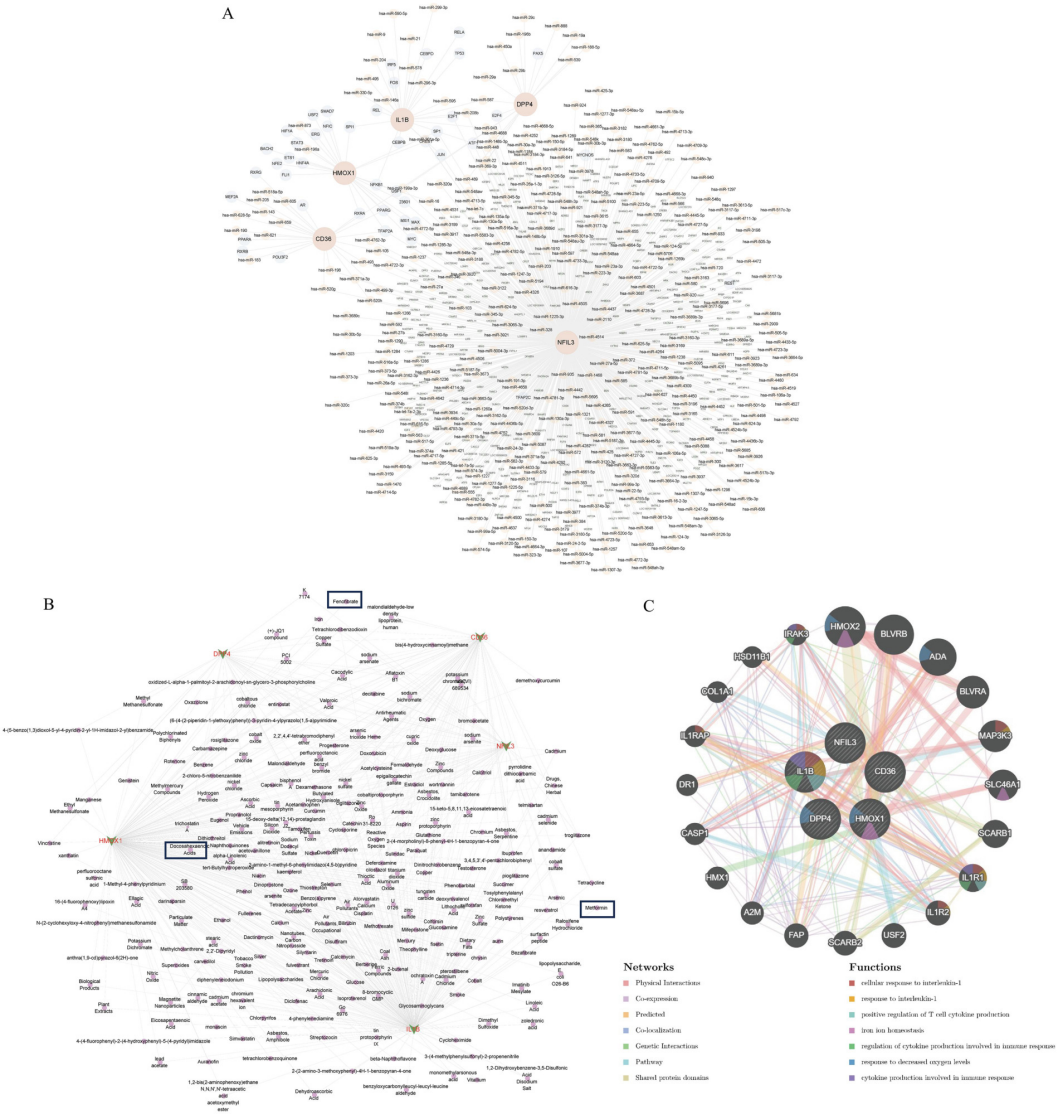
Gene-transcription factor (TF) regulatory network and protein-chemical interaction networks for model genes. **(A)** Model gene-TFs-microRNA regulatory network visualized in Cytoscape based on the regulatory relationships predicted by public databases. Red represents key diagnostic genes (hub genes), orange represents microRNAs, green represents protein-coding genes, and blue represents TFs. **(B)** Model gene-encoded proteins and chemical substances interaction network visualized in Cytoscape, displaying potential regulatory relationships of drugs or chemical molecules. **(C)** Protein-protein interaction network of key diagnostic genes based on the public protein interaction database, and functional annotation and pathway enrichment analysis of interacting proteins, revealing their potential biological functions.

To elucidate the regulatory mechanisms of key diagnostic genes, we constructed a transcriptional regulatory network using the NetworkAnalyst. The analysis revealed extensive interactions among five diagnostic genes and multiple TFs as well as miRNAs, forming an intricate regulatory network (Figure 6A). The diagnostic gene NFIL3 appeared as a central node within the regulatory network, exhibiting interactions with TF MYC and miRNA hsa-miR-27b. Furthermore, multilayered regulatory relationships were identified: DPP4 with hsa-miR-29c and PAX5; IL1B with hsa-miR-21 and FOS; CD36 with hsa-miR-205 and PPARA; and HMOX1 with hsa-miR-196a, STAT3, and HIF1A. These interactions suggest potential involvement in coordinated regulation of gene/miRNA expression, thereby influencing AS progression. Through the CTD, we predicted compounds that interact with these gene-encoded proteins. Notably, HMOX1 and IL1B demonstrated enriched interactions with compounds like Docosahexaenoic Acids, Fenofibrate, and Methotrexate, offering promising avenues for AS therapeutic intervention (Figure 6B). An interaction network built via GeneMANIA showed these genes are functionally enriched in processes such as response to interleukin-1, production iron ion homeostasis, and response to decreased oxygen levels (Figure 6C), indicating that they are not only interrelated, but also functionally synergistically participate in the inflammation and metabolic disorder process of AS.

### 3.6 HMOX1 promotes ox-LDL-induced dysfunction of foam macrophages by regulating ferroptosis

**Figure 7:**
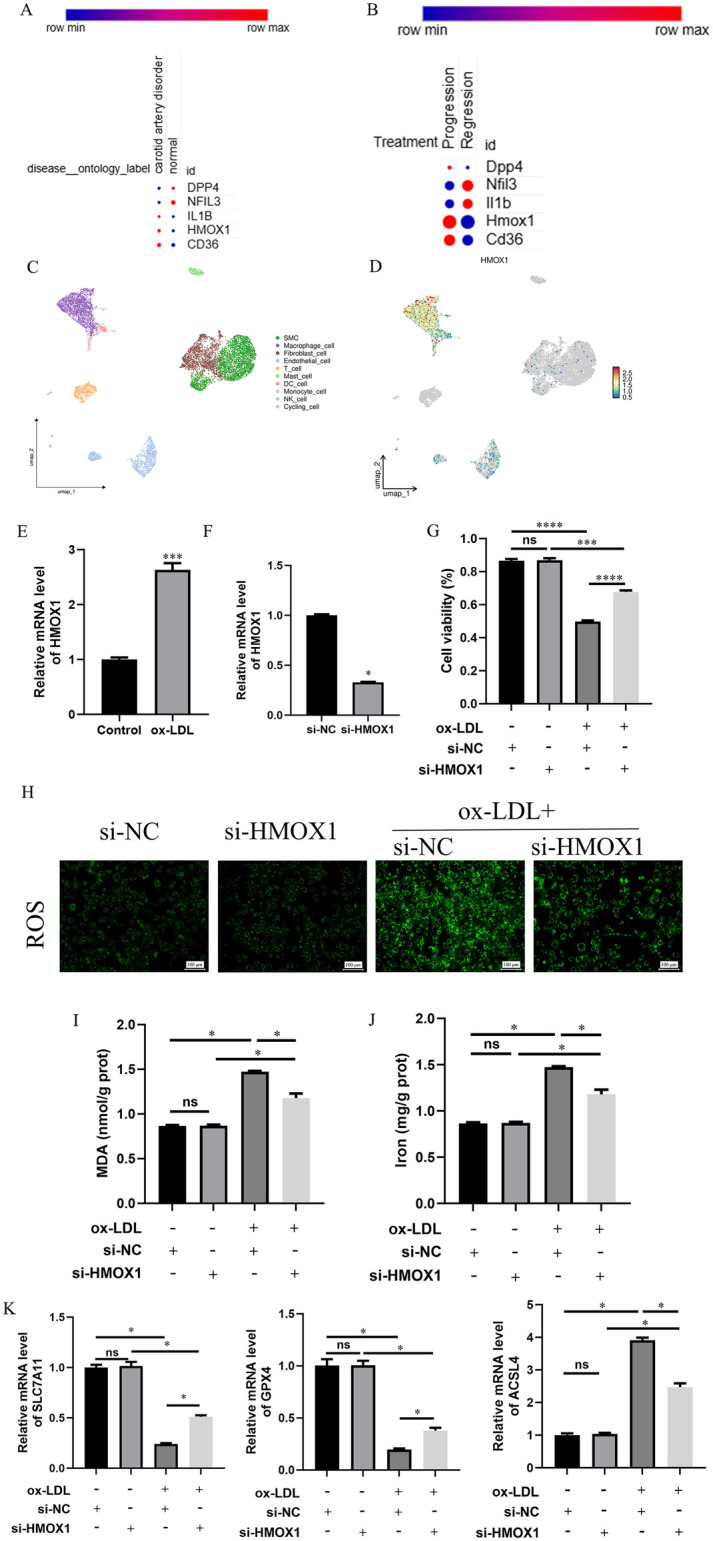
HMOX1 promotes ox-LDL-induced dysfunction of foam macrophages by regulating ferroptosis. **(A)** Expression of diagnostic genes in normal and AS groups. **(B)** Expression of diagnostic genes in AS progression and regression groups. The progression group and regression group were isolated from Cx3cr1^CreERT2−IRES−YFP/+^; Rosa26^floxed−tdTomato/+^ mice treated with AAV-mPCSK9 and subjected to dietary intervention. **(C)** Single-cell RNA sequencing data of the GSE155512 dataset, processed by the “Seurat” package and subjected to UMAP dimensionality reduction, showing the main cell type composition in the atherosclerotic samples. **(D)** Expression distribution of HMOX1 in different cell types. **(E,F)** Expression of HMOX1 in ox-LDL-induced THP-1 derived foam macrophages and verification of knockdown by qRT-PCR **(E)** and Western blot **(F) (G)** Effects of HMOX1 knockdown on macrophage viability under different conditions assessed by CCK-8 assay. **(H–J)** Effects of HMOX1 knockdown on oxidative stress **(H)**, lipid peroxidation **(I)**, and iron accumulation **(J)** induced by ox-LDL. **(K)**: Effects of HMOX1 knockdown on the expression of the key ferroptotic gene ACSL4 and anti-ferroptotic genes SLC7A11 and GPX4. All *in vitro* experiments were independently repeated three times. Continuous variables were expressed as mean ± standard deviation. Comparisons between two groups were performed using Student's *t*-test, and comparisons among multiple groups were evaluated using one-way ANOVA. Single-cell data analysis was based on R (version 4.4.2) and was mainly completed using R packages such as “Seurat” and “Harmony”. Statistical analysis and visualization were performed in R software and GraphPad Prism 10.1.2. ns: no statistical difference, **: *p* < 0.01, ***: *p* < 0.001, ****: *p* < 0.0001.

We further predict the expression patterns of five key diagnostic genes in AS and their progression. Results showed DPP4 and NFIL3 expression decreased, while IL1B, HMOX1, and CD36 increased in AS, compared with the normal group (Figure 7A). During AS regression, DPP4, HMOX1, and CD36 decreased after AS regression, while NFIL3 and IL1B increased (Figure 7B). HMOX1, a key enzyme in ferroptosis, drives ferroptosis by releasing free iron ions and triggering lipid peroxidation (32). Additionally, ferroptosis, an iron-dependent form of necrosis, contributes to AS development by promoting lipid peroxidation and oxidative stress (33).

To further elucidate the role of HMOX1 in AS, we performed scRNA-seq on the GSE155512 dataset, annotating cell types in AS tissues and identifying multiple cell types, including smooth muscle cells, macrophages, and endothelial cells. HMOX1 is predominantly expressed in macrophages (Figures 7C,D). Building on this, we used ox-LDL to induce human monocyte THP-1 cells into foam-like macrophages to investigate the function of HMOX1 in this model. qRT-PCR experiments revealed that HMOX1 expression was significantly upregulated after ox-LDL induction. Therefore, we knocked down HMOX1 using siRNA for subsequent functional validation (Figures 7E,F). The experiment was divided into si-NC, si-HMOX1, ox-LDL + si-NC, and ox-LDL + si-HMOX1 groups. ox-LDL treatment significantly inhibited cell proliferation, while HMOX1 knockdown partially reversed this effect, restoring cell proliferation capacity (Figure 7G). Furthermore, after ox-LDL induction, intracellular levels of ROS, MDA, and iron ions were significantly increased, whereas HMOX1 knockdown evidently reduced these indicators (Figures 7H–J). Regarding the expression of ferroptosis-related markers, ox-LDL treatment significantly decreased the mRNA expression of anti-ferroptotic proteins SLC7A11 and GPX4 while downregulating the pro-ferroptotic protein ACSL4. HMOX1 knockdown significantly upregulated SLC7A11 and GPX4 expression and downregulated ACSL4 expression (Figure 7K). In summary, HMOX1 is primarily expressed in macrophages during AS progression and influences ferroptosis in AS through oxidative stress, lipid peroxidation, and ferroptosis-related pathways.

## 4 Discussion

AS, a complex chronic inflammatory disease, is closely linked to lipid metabolism disorder and specific cell death patterns (34). This study systematically identified 17 AS-related DE-CM-FRGs by integrating WGCNA, differential expression analysis, CMRGs, and FRGs. Implementing three machine learning algorithms (LASSO, SVM-RFE, Boruta), we pinpointed five key diagnostic genes: CD36, DPP4, HMOX1, IL1B, and NFIL3. The logistic regression model based on these genes demonstrated strong discriminatory power in both training and validation sets, indicating high clinical translation potential.

CD36 accelerates AS progression by enhancing lipid accumulation, foam cell formation, inflammation, endothelial apoptosis, and thrombosis. Thus, inhibiting CD36 may be a novel strategy (35). Elevated DPP4 activity exacerbates AS under insulin resistance by boosting vascular inflammation, oxidative stress, and endothelial dysfunction. DPP4 inhibition alleviates inflammation and oxidative stress, improves endothelial function, and reduces AS lesions (36). IL1*β*, a pro-inflammatory cytokine, contributes to vascular inflammation; reducing IL1*β* levels mitigates vascular inflammation, thereby relieving AS lesions (37). NFIL3 holds a pivotal role in AS progression by regulating inflammation, macrophage polarization, immune cells, and lipid metabolism (38). Additionally, hydrogen sulfide inhibits MEST-mediated EndMT and attenuates low-shear-stress-induced AS by vulcanizing NFIL3 in endothelial cells (39). The aforementioned evidence supports the biological relevance of the genes screened in our study from the dimensions of inflammation, metabolism, and immunity.

Based on the bioinformatic screening results, HMOX1 was selected as a key gene for in-depth analysis. scRNA-seq revealed the enrichment of HMOX1 in macrophages within atherosclerotic plaques, indicating its importance in macrophage function. *In vitro* experiments confirmed significantly upregulated HMOX1 expression in ox-LDL-induced foam-like macrophages. Knocking down HMOX1 promoted cell proliferation, reduced ox-LDL-induced accumulation of ROS, MDA, and iron content, and upregulated anti-ferroptotic proteins SLC7A11 and GPX4 while downregulating the pro-ferroptotic factor ACSL4. These results suggest that in ox-LDL-stimulated macrophages, HMOX1 likely promotes ferroptosis, inhibiting proliferation, and increasing oxidative stress and lipid peroxidation, leading to AS progression. Numerous recent studies have gradually linked ferroptosis to AS onset and progression (12). Experimental research indicates that targeting the ferroptotic process can partially alleviate lipid peroxidation damage and endothelial dysfunction (13). Furthermore, impaired function of the key ferroptosis protective factor GPX4 has been closely associated with plaque formation and instability (14). We observed a trend of upregulated HMOX1 accompanied by downregulated GPX4 expression, aligning directionally with previous reports and suggesting the potential involvement of HMOX1 in ferroptosis-related regulatory networks. Regarding the underlying mechanisms, HMOX1 can enhance lipid peroxidation and increase cellular susceptibility to ferroptosis by promoting heme degradation and releasing free iron (40, 41). Moreover, HMOX1 might regulate ferroptosis in foam macrophages through its influence on the LDHB-mitochondrial function axis (42). These mechanisms align with the observations from our ox-LDL-induced model, though specific molecular pathways require experimental validation. We hypothesize that under oxidative stress conditions, such as ox-LDL stimulation, upregulated HMOX1 in macrophages may increase cellular sensitivity to ferroptosis by promoting iron accumulation and oxidative lipid damage; this hypothesis warrants mechanistic investigation. Overall, our results support an association between HMOX1 and changes in macrophage ferroptosis activity as well as AS-related phenotypes. HMOX1 may serve as a key node in the regulatory network, providing an experimental basis for future mechanistic research and therapeutic interventions.

In this study, we identified regulatory interactions between key genes and TFs/miRNAs, suggesting their pivotal roles in AS pathogenesis. NFIL3 emerged as a master regulator modulating multiple miRNAs, including TF MYC and hsa-miR-27b. Additionally, diagnostic genes exhibited associations with specific miRNAs such as hsa-miR-29c, hsa-miR-21, hsa-miR-205, and hsa-miR-196a. Mechanistic evidence reveals that MYC downregulation during vascular cell maturation suppresses splicing factors, changing circRNA levels. These changes are associated with the decreased level of circRNA in AS (43). In AS patients, miR-27b significantly decreases and independently correlates with multivessel disease severity, serving as a biomarker for systemic AS (44). miR-29c-3p upregulates in vascular smooth muscle cells (VSMCs), promotes the proliferation, migration, and apoptosis of VSMCs by inhibiting MYOSLID, thereby accelerating plaque formation (45). Moreover, enriched in endothelial colony-forming cell-derived exosomes, miR-21-5p, by inhibiting SIPA1L2 expression, repairs the autophagic flux, enhances autophagy activity, thereby promoting the proliferation, migration, and tube formation of endothelial cells, protecting vascular endothelium, and alleviating AS or vascular damage (46). miR-205-5p reduces ox-LDL-induced human aortic-VSMC viability, cycle progression, proliferation and migration, promotes apoptosis, and alleviates AS by inhibiting the phosphorylation of the ERBB4/AKT pathway (47). miR-196a-5p is upregulated in ox-LDL-stimulated VSMCs and aggravates AS (48). While these miRNAs are influential in AS, their specific regulatory relationships with our diagnostic genes remain unclear. Therefore, it warrants further validation for these regulating effects.

Through bioinformatic analysis, we identified chemical compounds that may interact with the diagnostic genes, thereby providing clues for potential therapeutic strategies for AS. As revealed by prediction, HMOX1 and IL1B demonstrated enriched interactions with compounds like Docosahexaenoic Acids, Fenofibrate, and Methotrexate. Docosahexaenoic Acids alleviate the self-induced cell stress and apoptosis by upregulating HMOX1 expression, suggesting that the upregulation of HMOX1 is a key negative feedback mechanism for its vascular protective effect (49). Fenofibrate upregulates HMOX1 via PPAR*α*, which is an important mechanism for it to exert anti-proliferative and anti-inflammatory effects and delay AS (50). Methotrexate exerts anti-inflammatory effects by inhibiting the production and release of the key pro-inflammatory factor IL1B in AS, thereby mitigating AS progression. Methotrexate inhibits IL1B production, exerting anti-inflammatory effects in AS (51). These findings deepen the understanding of drug mechanisms and provide a basis for future research targeting these pathways.

Although this study systematically screened out 5 key genes closely related to cholesterol metabolism and ferroptosis in AS through integrating WGCNA, multiple machine learning algorithms, and *in vitro* experiments, and successfully constructed a logistic regression diagnostic model with good discriminatory efficacy, limitations still exist. First, the transcriptomic data are only from public GEO databases, with a limited sample size and population diversity. Future validation with larger, independent, multi-center cohorts is needed. Secondly, based on the transcriptional features of lesioned tissues, our diagnostic model is currently positioned more as a tool for molecular subtyping and mechanistic analysis rather than a non-invasive diagnostic method ready to replace existing clinical tests. In potential clinical applications, the model along with its key gene signatures could serve as a supplement to conventional imaging and clinical risk assessments, helping to identify AS patient subgroups with specific metabolic reprogramming and ferroptosis abnormalities, aiding risk stratification and personalized treatment decisions. Future research could evaluate the expression correlation of these key genes in peripheral blood cells, circulating biomarkers, or other clinically accessible samples, and explore integrated diagnostic strategies that combine them with imaging features and clinical indicators, progressively translating them into clinically applicable tools. The key genes identified in our study demonstrate significant biological relevance through bioinformatic analysis and *in vitro* experiments, suggesting their potential as therapeutic targets or biomarkers. However, direct evidence regarding drug intervention efficacy and safety remains absent, and their clinical translatability requires further investigation. We also acknowledge that our strategy of intersecting WGCNA-derived module genes, DEGs, FRGs, and CMRGs has limitations; future studies could employ stratified analysis or network expansion approaches to explore the potential immune-regulatory functions of non-overlapping genes. In summary, expanding sample sizes, conducting multi-center and prospective validation studies, along with deeper functional and interventional experiments, will be essential to advance our findings toward clinical translation and to provide new theoretical foundations for the precise diagnosis and treatment of AS.

## Data Availability

The original contributions presented in the study are included in the article/Supplementary Material, further inquiries can be directed to the corresponding author.

## Supplementary material

Supplementary Table S1 **AS-related differentially expressed genes in the GSE100927 dataset (35 Control, 69 AS).** Differential expression analysis between AS and control samples was performed using the “limma” package, with screening criteria of |log₂FC| > 1 and *p* < 0.05.

Supplementary Table S2 **17 key genes related to AS in cholesterol metabolism and ferroptosis (DE-CM-FRGs) selected by Venn analysis**. The gene set was obtained by intersecting WGCNA-derived module genes, AS-related differentially expressed genes from the GSE100927 dataset (35 Control, 69 AS), cholesterol metabolism-related genes, and ferroptosis-related genes. All analyses were completed in R (version 4.4.2) using the “VennDiagram” package.
